# A Review of Numerical Models of Radiation Injury and Repair Considering Subcellular Targets and the Extracellular Microenvironment

**DOI:** 10.3390/ijms25021015

**Published:** 2024-01-13

**Authors:** Nousha Afshari, Igor Koturbash, Marjan Boerma, Wayne Newhauser, Maria Kratz, Jeffrey Willey, Jacqueline Williams, Jeffery Chancellor

**Affiliations:** 1 Department of Physics and Astronomy, Louisiana State University, Baton Rouge, LA 70803, USA; nafsha2@lsu.edu (N.A.); newhauser@lsu.edu (W.N.); 2 Department of Environmental Health Sciences, Fay W. Boozman College of Public Health, University of Arkansas for Medical Sciences, Little Rock, AR 72205, USA; ikoturbash@uams.edu; 3 Division of Radiation Health, Department of Pharmaceutical Sciences, College of Pharmacy, University of Arkansas for Medical Sciences, Little Rock, AR 72205, USA; mboerma@uams.edu; 4 Department of Biological and Agricultural Engineering, Louisiana State University, Baton Rouge, LA 70803, USA; mkratz3@lsu.edu; 5 Department of Radiation Oncology, Wake Forest School of Medicine, Winston-Salem, NC 27101, USA; jwilley@wakehealth.edu; 6 School of Medicine and Dentistry, University of Rochester Medical Center, Rochester, NY 14642, USA; jackie_williams@urmc.rochester.edu; 7 Department of Preventive Medicine and Population Health, University of Texas Medical Branch, Galveston, TX 77555, USA; 8 Outer Space Institute, University of British Columbia, Vancouver, BC V6T 1Z4, Canada

**Keywords:** radiation, nuclear DNA, radiobiology, mitochondrion, GCR, DNA damage, DNA repair mechanisms, mitochondrial DNA, organelles, HZE

## Abstract

Astronauts in space are subject to continuous exposure to ionizing radiation. There is concern about the acute and late-occurring adverse health effects that astronauts could incur following a protracted exposure to the space radiation environment. Therefore, it is vital to consider the current tools and models used to describe and study the organic consequences of ionizing radiation exposure. It is equally important to see where these models could be improved. Historically, radiobiological models focused on how radiation damages nuclear deoxyribonucleic acid (DNA) and the role DNA repair mechanisms play in resulting biological effects, building on the hypotheses of Crowther and Lea from the 1940s and 1960s, and they neglected other subcellular targets outside of nuclear DNA. The development of these models and the current state of knowledge about radiation effects impacting astronauts in orbit, as well as how the radiation environment and cellular microenvironment are incorporated into these radiobiological models, aid our understanding of the influence space travel may have on astronaut health. It is vital to consider the current tools and models used to describe the organic consequences of ionizing radiation exposure and identify where they can be further improved.

## 1. Introduction

Understanding the risk to astronaut health from exposure to the space radiation environment, including that from high-energy and high-charge particles (HZE), has been a priority since the beginning of the National Aeronautics and Space Agency’s (NASA’s) human spaceflight endeavors [1]. The magnitude of radiation exposures and the corresponding effects vary enormously, from a negligible increase in cancer risk after the mission to in-flight death from acute radiation syndrome. For many reasons, space-based research has limited capability to ascertain magnitudes, explain mechanisms, or predict the occurrence of adverse health outcomes in humans [1]. Alongside experimental radiobiology, numerical models aimed at describing radiation effects in humans were developed from a study where simplified cells composed of deoxyribonucleic acid (DNA), cytoplasm, and cellular membrane were irradiated with gamma- or x-rays [2]. These studies broadly concluded that DNA was the more radiosensitive structure of the two intracellular compartments and the primary target of relevance to radiation-induced biological effects, excepting hereditary effects. More recent evidence suggests there are multiple biological targets within the cell, but this early experimental evidence altered the trajectory of radiobiological model development [3,4]. Cellular models based solely on DNA damage and cell death do not entirely explain or even agree with some experimental data, particularly for acute and late effects. A likely explanation for these limitations is that non-nuclear subcellular structures are important but have not been explicitly considered yet [5]. Furthermore, there is a need to integrate modeling of key mechanisms of biological action, including those at the subcellular, cellular, tissue, and organism levels [6].

Many acute and long-term effects can arise from exposure to ionizing radiation. In addition to tissue and organism level effects, such as cognitive impairment, acute radiation syndrome, and degenerative tissue diseases, there has been extensive research focused on cellular and subcellular level radiation exposure effects. The tools used in risk stratification regarding these conditions and outcomes are limited and could be improved upon [7,8]. The National Council on Radiation Protection and Measurements (NCRP) identifies three primary health risk concerns for long-term missions outside Earth’s magnetic field: cancerous late effects, noncancerous early effects, and possible effects on the central nervous system from HZE particles [7]. The risk is calculated using equivalent dose and a tissue-specific risk coefficient, both of which are estimations [7]. Equivalent dose is obtained from relative biological effectiveness (RBE)-derived radiation weighting factors for latent or stochastic effects, and the tissue risk coefficient is approximated from the shielding distributions at different points within each organ [7]. The radiation-related cancer risk is well studied and can be quantified with some uncertainty. In comparison, the relationship between low dose rate thresholds, like that seen beyond the low Earth orbit (LEO), and the risk of long-term noncancerous effects occurring is not well-defined. The regulatory bodies of space radiation protection and safety acknowledge the limitations of the conclusions made and note that the late biological effects of radiation are unknown and need further study [7].

With treatment planning in the clinical setting, extra care is applied to delivering the maximum dose to a tumor while simultaneously minimizing the dose to the surrounding tissue (i.e., critical organs or tissue structures) [6]. Clinical tools that provide risk stratification of radiation effects are not yet applicable in assessing astronaut health risks from the spaceflight environment [1]. Thus, there are limitations to determining the dose and dose rate thresholds for noncancerous late biological effects.

To date, there is limited evidence of long-term nonmalignant pathologies manifesting in humans who have flown in space that can be directly attributed to an exposure to the space radiation environment [9]. Focusing on nuclear DNA damage and repair mechanisms fails to fully characterize the long-term effects of radiation. Here, we will review the historical development of radiobiological models, the factors affecting radiation sensitivity and resistivity within the cell’s microenvironment, and several recent advancements in the radiobiology field, including the role of mitochondria and nuclear DNA damage and repair, and their involvement in radiation response. We will also briefly expand on the epigenetic elements involved in radiation effects outside of hereditary factors.

## 2. Background

### 2.1. Space Radiation Environment

The space radiation environment in LEO consists of four primary sources: solar wind, solar particle events, Galactic Cosmic Rays (GCRs), and trapped particles in the Van Allen belts [9]. Outside of LEO, GCRs are the primary concern since they are part of the normal radiation environment and are difficult to completely mitigate with spacecraft shielding. The GCR spectrum consists of relativistic, fully ionized heavy charged particles originating outside the Earth’s solar system. The GCR spectrum is composed of approximately 87% hydrogen ions, 12% helium ions, 1% electrons, and 1% HZE [4,10]. The HZE contribution to the GCR spectrum ranges from lithium (Z = 3) up to nickel (Z = 28), with a significant contribution to biological damage to living organisms coming from iron (Z = 26) [9,10]. Shielding against these HZE particles can lead to potential nuclear reactions within the spacecraft material, generating cascades of secondary particles [7]. These secondary particles can then increase astronaut exposure and may confer more risk than the primary radiation [7].

**Figure 1:**
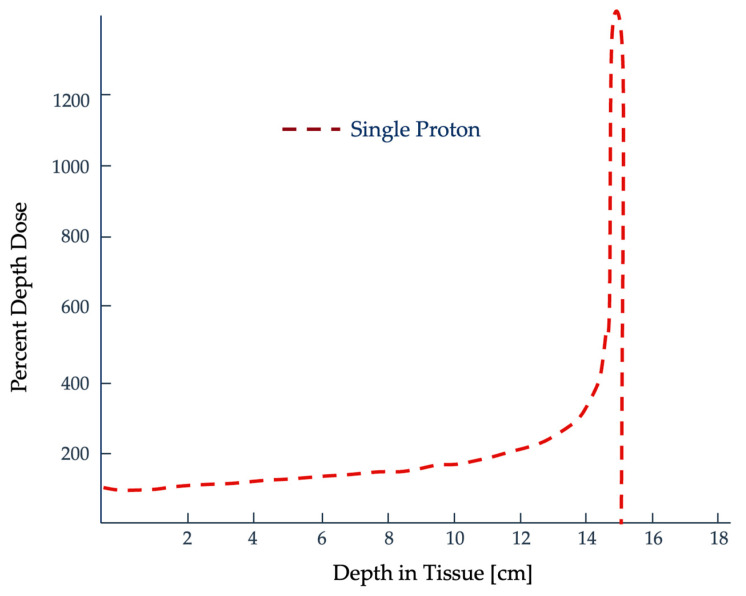
This maximum dose deposition is referred to as the Bragg peak, which is used advantageously when treating patients with heavy charged particles [13]. Graph adapted from Wilson [14].

Heavy ion exposures play a critical role in astronaut spaceflight risk assessment. They have a finite range within tissues with minimal dose deposition until the end of the particle track, where nearly all of their energy is delivered [11]. As a heavy ion barrels through a medium, it loses energy continuously with each interaction, which can impart biological damage [9]. Most of its energy is deposited within a short distance at the end of its path. Figure 1 shows what is referred to as the Bragg peak, which is used advantageously in radiotherapy [9,12,13]. In terms of biological effects, heavier ions (Z > 3) like carbon are more effective at creating irreparable DNA damage and their efficacy is not dependent on the presence of oxygen, which is a significant advantage when treating radioresistant hypoxic tumors [11].

### 2.2. Quantifying Radiation Damage

A radiation particle can interact with its environment to lose all or some of its energy into the medium. If the energy is great enough, an electron of the medium’s atom can be ejected from its orbital shell. The atom is then categorized as ionized and can continue interacting with the medium and cause damage. There are two ways for radiation-induced damage to occur: directly and indirectly. Direct action occurs when the projectile reacts with the target (e.g., DNA). Indirect action refers to a particle that hits near the target in the microenvironment, generating free radicals, or ionized atoms, that chemically react with the target [15]. Free radicals are atoms or molecules with an unpaired orbital electron, making them highly chemically reactive. The spatial energy distribution between x- or gamma-ray and heavy-ion irradiations are very different. Photons are randomly distributed across the cellular volume and as a result the ionization density is assumed to be homogenous [16]. In contrast, heavy ion spatial distribution of energy is more localized, which results in more damage to the volume and lower probability of repair (primarily DNA-strand break repair), thus having a larger biological effect [16].

Linear Energy Transfer (LET) describes the energy a particle transfers per unit length of a track as it traverses a medium [2]. High-LET particles, such as carbon, are more densely ionizing along their paths than low-LET particles, e.g., secondary electrons liberated by x- or gamma-rays [12,17]. This can result in more damage produced within the medium through high-density clusters of ionization and biological damage. Research suggests that direct action damage is the dominant process responsible for space radiation exposure’s more concerning biological effects related to DNA damage and the probability of cell death or misrepair of strand breaks.

The cellular response following irradiation depends on how the average LET is specified. There are two ways: track average and energy average LET. For track-averaged LET, one divides the particle’s path into segments of equal length, then reports the average energy transferred within a segment. For energy-averaged LET, one partitions the path length into equal energy loss increments and then reports the mean of the iso-energy loss path lengths [2,18]. The choice of the average LET used can sometimes make a big difference. While both averages yield similar results with x-rays and monoenergetic charged particles, neutrons are better described by the energy average LET [2]. Furthermore, as LET increases, the variability of an ionizing particle’s lethality across the cell’s cycle decreases so that radiosensitivity appears independent of the cell cycle at higher LET [19]. In space, astronauts are exposed to continuous high LET radiation environments at low fluence rates (i.e., low numbers of particles per area of interest) for protracted periods. The radiobiological tools used to describe the long-term effects of protracted low-dose exposure are limited, especially when definitions such as average LET are inconsistent across different experimental and research analyses.

**Figure 2:**
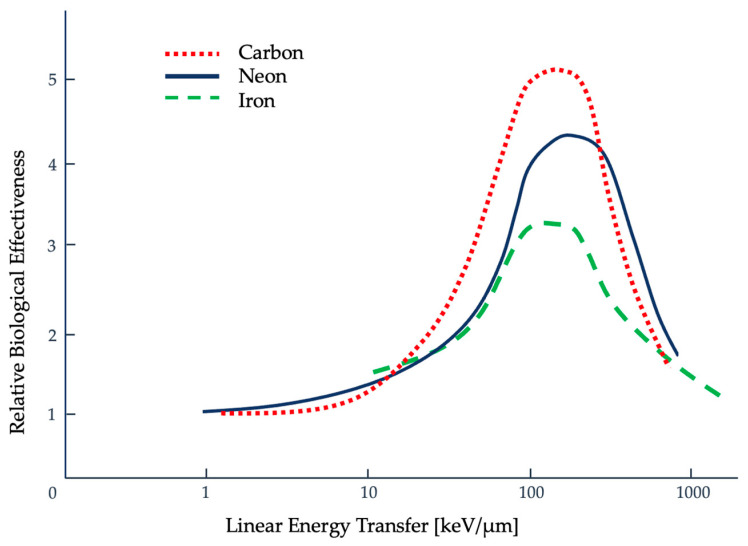
Relationship between relative biological effectiveness (RBE), clonogenic cell death, and LET for mammalian cells with carbon, neon, and iron [2,23,25]. Here, it can be seen that around 100 keV/micron, along the LET axis, there is a peak after which the RBE not only fails to increase but declines. Figure adapted from Sørenson [25].

Another method used to describe the efficacy of a radiation type is the RBE, which is the ratio of absorbed dose of one type of radiation to a specified, standard x-ray radiation (e.g., 250 kVp x-rays) to produce the same biological effect [20,21]. In the field of radiation protection, the estimation of the biological response uses weighting factors that are dependent on the radiating particle type and the radiosensitivity of the organ the particle is traversing [16]. These weighting factors, also referred to as quality factors, are upper limits that overestimate radiation effects assigned by the International Commission on Radiological Protection (ICRP) [16,22]. The trend for RBE against high-LET radiation of heavy ions, based on the clonogenic death of mammalian cells irradiated in culture, initially shows a positive correlation to about 100 keV/micron. Past this threshold, an inverse relationship is then seen, as shown in Figure 2, and has been linked to double-strand DNA breaks [23]. This is referred to as the overkill effect, or overkill phenomenon where additional energy is wasted [24]. Figure 2 shows how the RBE maximum varies with particle type, but the overkill effect can be seen to occur at approximately 100 keV/μm [25].

This simplistic definition of RBE, the “same biological effect”, can refer to the likelihood of a particle’s lethality and the probability of it producing nuclear DNA double-strand breaks [24]. RBE can also be a measurement used to describe nonlethal radiation effects outside of DNA double-strand breaks, within the context of space radiation. Nevertheless, the uncertainties are significant and increase directly with increasing RBE [26,27,28]. The error within RBE is due to the variability in stochastic processes (i.e., those involved in measuring cellular damage) and radiosensitivity (i.e., affected by cell type, radiation type, and microenvironment). Despite empirical evidence, RBE is roughly estimated because of these factors [28]. An error that is too large in the RBE allocation can result in an underdosage or overdosage of the tumorous or normal tissues [29]. The guidelines for RBE are partly set by the International Commission on Radiation Units and Measurements (ICRU) and by a country’s legal limitations [29]. ICRU Report 78 requires that the contribution of the error to the prescribed dose by RBE falls within −5% to +7% and that an RBE of 1.1 is used for proton therapy at all dose levels, regardless of the factors present contributing to the variability [29].

In 2008, Cucinotta et al. conducted a study measuring the organ dose equivalent of astronauts aboard the International Space Station (ISS) [30]. They reported that about 80% of the radiation contribution was from GCRs and the uncertainties in radiation outcomes are compounded by how broadly radiation quality and dose rate effects are determined [30]. The average effective doses for astronauts aboard the ISS, where exposures were modified by the shielding from the spacecraft’s walls, did not consider extravehicular activities in the data analysis.

RBE is an essential in heavy-ion radiotherapy treatment planning, focusing on tumor control and minimizing damage to the surrounding normal tissues. “Clinical RBE” describes the ratio of a prescribed absorbed dose between a photon and a high-LET radiation particle to result in clinically equivalent outcomes [31]. Heavy-ion irradiation is empirically found to be more biologically effective when compared to the same absorbed dose between particle types. Clinical RBE values are adjusted based on medical expertise and decisions because the RBE within the tumor volume may vary [16,31]. RBE within an ion beam varies and is compensated for by modulating the absorbed dose from RBE model predictions. Thus, clinical RBE is implicitly determined from the model used and the input parameters of the radiation environment. Experimentally, RBE can be measured based on the specific irradiation conditions, but these same conditions do not hold true within a patient [31]. Between acute reaction (i.e., caused by cellular depopulation) and late reaction (i.e., from chronic inflammation) tissues, proton RBE data from in vitro and acute-reaction in vivo experiments are more likely to underestimate RBE in late-reacting tissues [29].

Incorrect dosages given in the clinic can have significant biological consequences, especially on the probability of causing late-developing malignant (i.e., cancerous) or nonmalignant (e.g., progressive fibrosis, vascular insufficiency, etc.) diseases in treated patients [29]. This is also a concern in the realm of space radiation exposure. The technological advancements in treatment planning at the time the guidelines were developed were not able to easily adjust for a variable [29]. Instead of use as a definitive indication of whole-organ or whole-body nonmalignant pathological outcomes, RBE is better utilized to describe the frequency and presence of lesions created by DNA strands from ionizing radiation that result in the cell culture’s inability to continue proliferating and eventual death [27].

In the following section, the concept of a “sensitive volume” and the assumptions made regarding radiation effects will be discussed in the context of radiobiological model development. Based on the simplified experiment that determined nuclear DNA as the primary target, or “sensitive volume”, of ionizing radiation, there is a variability in experimental results that has yet to be well defined. The error in radiation effects and RBE seen clinically and experimentally could stem from this lack of definitive evidence that there is only one target, and it is nuclear DNA. This possibility that there could be multiple sensitive volumes that are responsible needs to be further investigated.

### 2.3. Radiobiological Numerical Models

The cell is the fundamental building block of human tissues and organs. Development of the first models describing the biological effects seen following ionizing radiation exposure began before the “sensitive volume” within the cell was identified. Soon after the first radiographic image was taken, scientists attempted to model and explain the physiological, biological, and chemical phenomena at the subcellular level following irradiation of an organic medium [6]. The first application of target theory was developed in 1924 by Crowther and improved upon by Lea. Target theory dominated the field of radiobiology until the 1980s and had two subcategories: the Single-Target Single-Hit (STSH) model developed by Crowther and the Multi-Target Single-Hit (MTSH) model from Lea [32]. The term “hit” refers to the ionizing radiation particle interacting with the medium and depositing dose into the sensitive volume within the target cell. Crowther found an exponential loss in biological activity following exposure to ionizing radiation [33,34]. This biological activity, also called cell survival, refers to the cell’s ability to continue proliferating after irradiation, and is given by

(1) $$S=S_{0}e^{-IV},$$

with $S_{0}$ for the initial percentage of viable cells, *V* as the “sensitive volume”, and the ionization density, *I*, determining cell survival. An exponential relationship between radiation exposure and cell survival was expected. It was assumed that the administered radiation would enter the sensitive volume *V* and inactivate the cell. Crowther’s method used roentgens, a unit that better described the ionization of air particles and did not hold for condensed media [33]. Eukaryotic cells are composed of organelles communicating with the cell’s internal and external environments. Exposure, or the ionization of air, was a reasonable quantity for the intent and purpose of Crowther’s experiments, but the quantities defining air effects are insufficient to describe complex biological damage.

Lea extended the sensitive volume concept within target theory with the (MTSH) model. He theorized the inactivation of the tested organic samples was related to the formation of lethal mutations and that there were multiple targets within *V* [35,36]. From irradiating bacteria and viruses, he made assumptions that cell killing was a multi-step process: there needed to be an absorption of energy within a sensitive volume; lesions in the cell were created by energy deposition, and in a subsequent step, these lesions resulted in the cell’s inability to proliferate [5,35]. Target theory models successfully described radiation effects in some microbiological systems but failed to describe radiation effects seen in higher plant types and mammalian samples [5,27].

**Figure 3:**
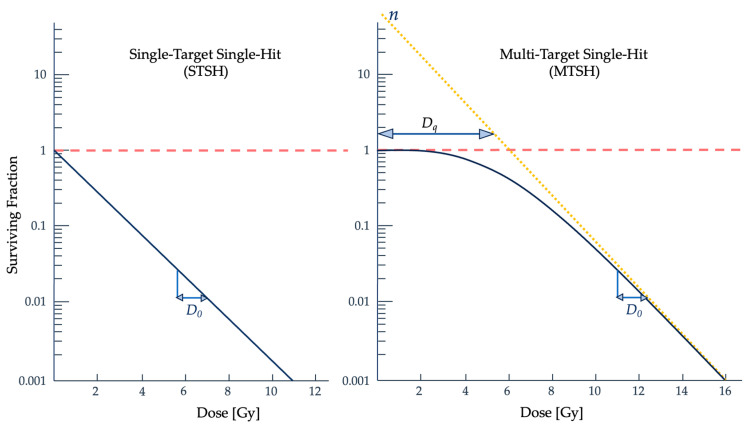
Comparison of the STSH and MTSH models [12]. *D_0_* describes the slope of the curve’s linear portion, and *D_q_* gives the approximate dose range, or width, of the curve’s shoulder. A linear slope on these semi-logarithmic graphs describes an exponential relationship. The STSH model, shown on the left, was expected to be seen, but a shoulder would appear in the data instead, depicted on the right. Graphs adapted from Joiner and Kogel [12].

Experimentally, it was noted that more complex cells had a higher radiosensitivity than bacteria and viruses and that an initial dose to a sample does not always result in an exponential relationship with clonogenic cell survival. The range of dose where there is a delay in lethal damage to the cell is known as the quasi-threshold dose (*D_q_*) and describes the “shoulder” of the cell survival curve, which can be seen in Figure 3 [12]. Building from the fundamental assumptions that each ionization causes damage to the molecular structure, Lea derived an equation that involved the molecular mass instead of a volume and *D_q_*, which together describe the shoulder portion of the survival curve [33]:

(2) $$S=S_{0}e^{-DM/N}$$

where *D* is the dose, *M* is the molecular mass, and *N* is the number of hits within the target. This model assumed that no “hits” meant cell survival, that each target had an equal probability of being hit by ionizing radiation, and that a single hit was enough to inactivate the target [32,35]. The MTSH model appropriately follows experimental data in high dose ranges and is described by single- or multi-event killings, shown by the curved and linear portions of the curve, respectively [35].

Radiosensitivity may vary depending on the period in the cell cycle when the radiation is received, the dose rate, and the microenvironment where the reactive oxygen species (ROS) are created [2,37]. Cell proliferation occurs via a cycle of mitosis, cell division, and DNA synthesis [38]. Since radiosensitivity is independent of the cell cycle at higher LET, the models used to explain cell reactions to ionizing radiation are limited to abnormal tissue behaviors, such as the cancerous cells, which have a faster proliferation rate—again, cementing these models’ application in the clinical setting [23]. The single- and multi-target models’ limitations are that they do not match experimental data in the lower dose range. It was expected that there would not be a shoulder to the curve in a lower dose range, whether for radiotherapy or space radiobiology purposes, but there is.

Chadwick et al., 1973, were some of the first to incorporate subcellular components into the numerical modeling [19]. This approach was termed the molecular theory and encompassed a broad class of radiobiological models considering subcellular processes in a cell’s reaction to irradiation. This model allowed insight into the radiobiological variability seen in irradiation experiments and assumed that damage to the critical structures within the cell affecting reproduction was to the double-strand nuclear DNA [19,39]. It was hypothesized that what was seen experimentally was due to the cell’s ability to repair DNA damage from irradiation and combat the lethality of the administered dose. Single-strand breaks would be appropriately repaired, while double-stranded DNA damage would likely lead to permanent cellular damage [19]. The purpose of the molecular theory model, shown in Equation (2), was to connect the physical and biochemical experimental observations; however, it bypassed several intracellular molecular functions and focused on repair mechanisms specific to nuclear DNA [39].

(3) $$S=S_{0}e^{-pf_{0}(n_{0}k_{0}∆D+\varepsilon n_{1}n_{2}f_{1}f_{2}k^{2}{\left(1-∆\right)}^{2}D^{2})}$$

Here, *p* is the proportionality factor connecting DNA double-strand breaks to cell death, *f* is the proportion of DNA double-strand breaks not repaired, *n* is the number of sites, *k* is the dose per site needed to result in double-strand breaks, Δ represents the probability of a single event double-strand break, *ε* is the proportion of bonds broken that are DNA double-strand breaks, and *D* is the dose administered.

**Figure 4:**
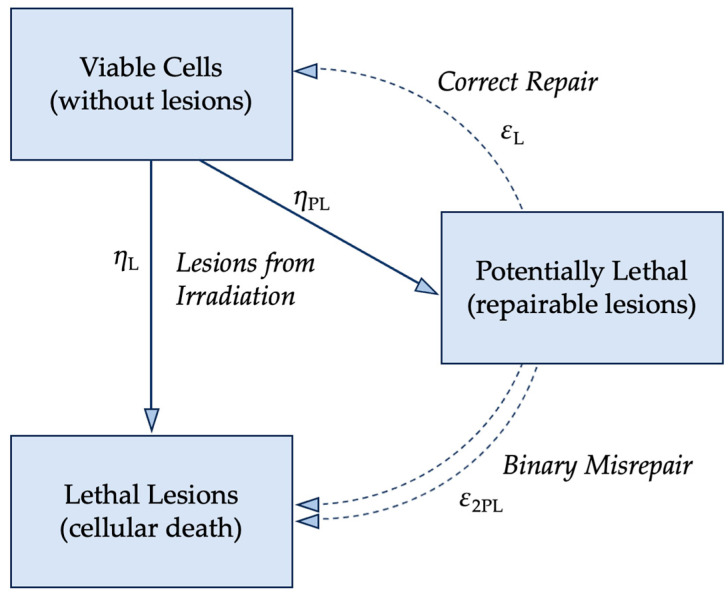
The LPL model was built on the assumption that the cell’s repair rate of DNA strand breaks is fixed and that the dose rate can be variable [40]. *η* represents the implicit dose rate and *ε* is the repair rate. Dose to the viable cells could produce potentially lethal (*PL*) lesions which could be repaired, but if misrepaired, or if the repair is not fast enough to combat the rate of lesion production, then the *PL* lesions can become lethal lesions and result in clonogenic cell death. If the repair rate is greater than the dose rate, and if any misrepairs do not impede the cell’s ability to proliferate, then the *PL* lesions are resolved, and the cell returns to its viable state [12]. Figure adapted from Joiner et al. [12].

The lethal–potentially lethal (LPL) model formulated by Curtis further built upon the foundation that nuclear DNA is the primary target of ionizing radiation. Figure 4 visualizes the LPL model with *η* as the implicit dose rate and *ε* as the repair rate, which is assumed constant; the repair mechanisms work at a fixed rate regardless of the concentration of damaged DNA. Potentially lethal (*PL*) lesions may be repaired and return the cell to the viable, pre-damaged state, or become lethal (*L*) lesions that result in cell death. The numerical description of the LPL model is shown in Equation (3). While *ε* and *η* are implicitly the repair and dose rates, respectively, *t_r_* is the total repair time available after exposure to ionizing radiation.

(4) $$S=S_{0}e^{-\{\left(\eta_{L}+\eta_{PL}e^{\varepsilon_{L}t_{r}}\right)D+\left(\frac{{\eta_{PL}}^{2}}{2\varepsilon }\left(1-e^{-\varepsilon_{L}t_{r}}\right)D^{2}\right)\}}$$

**Figure 5:**
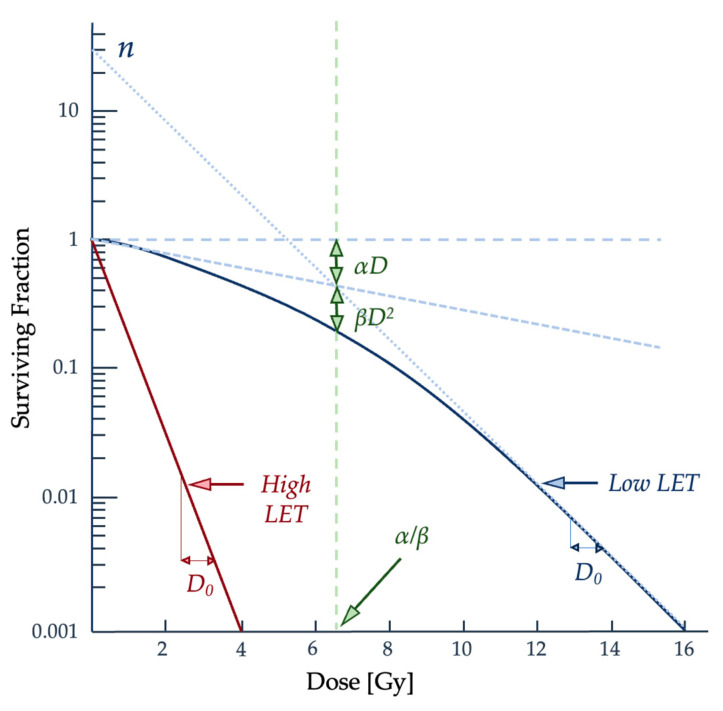
The linearity of the curve on the logarithmic–linear scale represents an exponential relationship between the dose and the surviving fraction [12]. Densely ionizing radiation, or high LET particles such as α particles and neutrons, is the right-hand curve shown in red and is more likely to result in a linear curve. Sparsely ionizing radiation or low-LET particles such as x-rays will produce more of a shoulder to the curve, as described by *D_q_* [2,12]. Figure adapted from Hall and Giaccia [2].

Combining the probabilities and concepts within molecular theory and the LPL model, the linear quadratic (LQ) model was subsequently derived. This model has several limitations that have continued into the development of modern numerical models. Although these models hold for the irradiation of individual cells in vitro and in vivo, there are limitations to the validity of these models at low dose rates [12]. Furthermore, the time dependence is implicit and may only describe the presence or absence of cell inactivation [12,41]. The experimental data described by this model was obtained from yeast, bacterial, and viral samples irradiated in vitro and is defined by the following equation:

(5) $$S=e^{-(\alpha D+\beta D^{2})}$$

with *S* as the percentage of irradiated cells that can continue proliferating and *D* for the total radiation dose administered [32,36]. The αD component describes the single hits on the DNA strands, while the βD^2^ term describes multiple hits [32,36]. The behavior of the LQ model equation should result in a continuously curving relationship, which does not match experimental data for prolonged radiation dosage since there is a linear portion to the curve [36]. The curve in Figure 5 shows the relationship between the dose given and the resulting proportion of surviving cells and the difference in radiation effectiveness for clonogenic death at varying dose levels and particle types.

**Figure 6:**
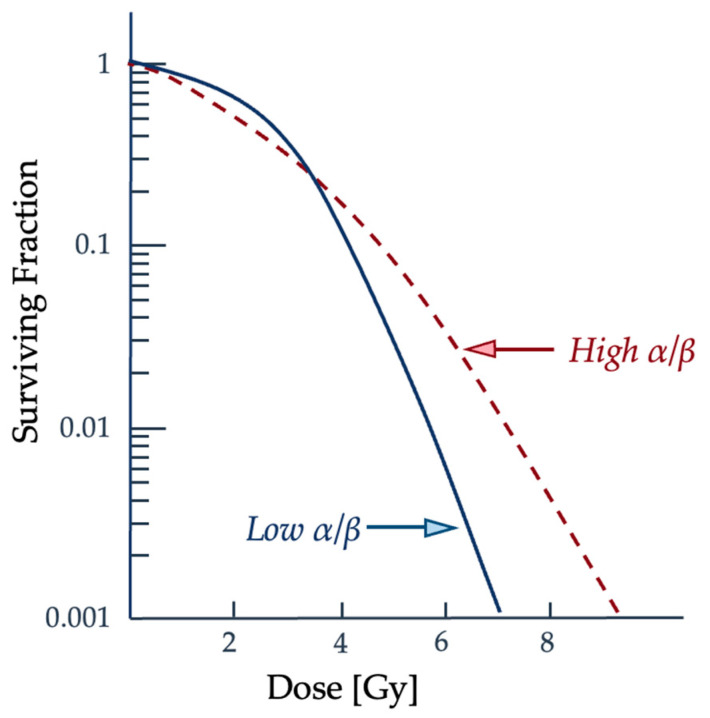
As the dose increases, the surviving fraction decreases, but the severity and concentration of double-strand breaks are variable between radiation types and cell lines. The lower an α/β ratio is, or higher the particle’s LET, the more likely double-strand breaks from a single particle interaction will occur when it traverses the biological medium [36]. Graph adapted from McMahon [36].

The α term is also used to refer to lethal damage from a single particle and the β term is reserved for the accumulating lethal damage caused by more than a single particle track [36]. The α/β ratio corresponds to the dose at which each type of damage is equal and is often used to characterize the radiosensitivity of cell lines. Low α/β ratio cell lines have a more pronounced curvature to the cell survival graph, while higher α/β ratio cell lines show a more constant rate of cell-killing as the dose increases [36]. How a cell survival curve, or response curve, is produced and whether the cell line is “high α/β” to “low α/β” is contingent upon the radiation conditions and potentially the microenvironment and type of cell. Changes to the cell cycle and target environment for low-LET radiation have been shown to cause shifts in the cell cycle and change a cell population from “high α/β” to “low α/β” [36]. This comparison of how different cell line types may result in a difference in the resulting cell survival curve can be seen in Figure 6.

As the dose increases, the surviving fraction decreases, but the severity and concentration of double-strand breaks vary between types of radiation and cell lines. The α/β ratio describes the type of damage the ionizing radiation is capable of at each dose in relation to the cell line type and is reflected in the curvature of the graph [36]. The α term is defined as the probability of cell death from a single incident particle causing lethal damage, while β reflects the probability of lethality from multiple hits [36]. It should be noted that cell survival curves for individual cells or asynchronous cell populations differ from the irradiation behavior of synchronous cells such as tissue. As a result, the LQ model has very low accuracy when describing impacts on cellular systems.

Further evidence is needed to support how damage to subcellular structures beyond nuclear DNA, the cell’s type, or cellular microenvironment can alter the α/β ratio of a cell population. More models emerged from the LQ model to explain the biological phenomena of cell survival and to try and improve the accuracy, such as the repair–misrepair (RMR) model, the saturable repair model, the two-lesion kinetic model, and the repair–misrepair–fixation model [32,35]. These models failed to directly link radiation damage to double-strand breaks for all radiation and cell line types [42].

The two-lesion kinetic (TLK) model aimed to connect the biochemical processes of double-strand break repairs with an ionizing radiation’s lethality [42]. This model considers the variability in cellular DNA repair mechanisms and that these repair systems saturate the microenvironment at higher dosages. The TLK model also differentiated between the two types of DSBs and, while it is similar to the LPL and RMR models, it accounts for the local complexity of the damaged site [42]. It can incorporate more parameters into its formalism, allowing for better agreement with experimental data and introducing additional complications.

The repair–misrepair–fixation (RMF) model combines the LPL and RMR models with microdosimetry concepts into a predictive model [43]. By this combination, the RMF model considers the intra- and inter-track binary misrepairs of DSBs and relates this damage to RBE [44]. However, as previously discussed, RBE is inadequate for describing radiation effects beyond the particle’s lethality. Furthermore, the RMF model assumes that the interactions of ionizing radiation with nuclear DNA resulting in double-strand breaks affect the nucleus as a whole [43].

There is a continued attempt to incorporate more modern tools into the radiobiological models. The Monte Carlo damage simulation (MCDS) has been combined with the RMF model, dosimetric data, and a Monte Carlo radiation transport model to improve the formalism for cell survival prediction [45,46]. This method can predict some double-strand and single-strand break and repair behaviors and can be applied to hypoxic microenvironments with differing types of ionizing radiation. Additionally, the local effect model (LEM) and the microdosimetric kinetic model (MKM) are two that aim to directly correlate energy deposition and subsequent cellular effects [36]. These models were developed to connect the deposited energy with the radiation-induced biological effect, though their usage remains primarily limited to radiotherapy. The field of space radiobiology currently relies on the LQ model, and the potential for applying these newer models has yet to be seen. Importantly, radiobiological models are built on the hypotheses of Crowther and Lea and the common assumption that nuclear DNA is the only target of concern when studying radiation effects. However, if this were the case, it can be argued that the survival curves of different cells should be very similar in shape and slope.

**Figure 7:**
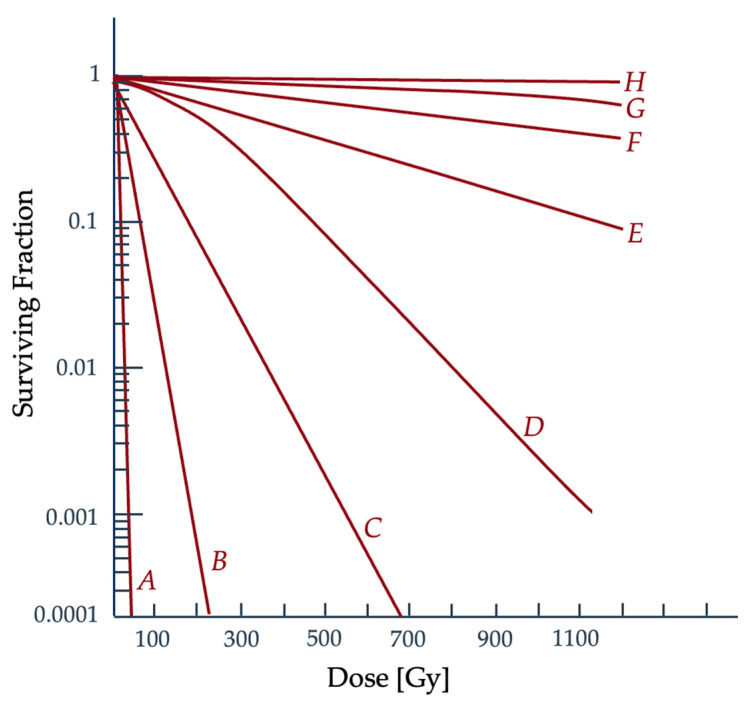
Comparison of different types of cells and their response to ionizing radiation. Shown is a comparison of (A) a mammalian cell line radiation response curve with that of (B) *E. coli*, (C) *E. coli* B/r (a mutation of *E. coli*), (D) yeast, (E) phage staph E, (F) bacillus megaterium (G) potato virus, and (H) M. radiodurans (one of the most radioresistant known organisms) [2,49]. Figure adapted from Hall and Giaccia [2].

In vitro radiation studies of irradiating cancerous and noncancerous mammalian cells have confirmed higher radiosensitivity than bacteria and viruses, shown with an increased slope in their survival curves [47]. The resulting survival curve comparison between cell types can be seen in Figure 7, where there are distinct slope and shoulder width differences. While prokaryotic cells lack distinct nuclei and organelles, eukaryotic cell structure is much more complex [48]. Mammalian cells have a nucleus and most contain mitochondria with mitochondrial DNA, as well as other large subcellular structures. Since the radiosensitivity of mammalian cells is greater, there could potentially be additional targets within the cell outside of nuclear DNA, which is a theory in need of further investigation [47].

Contributors to the early models stated that more survival curve analyses were necessary to prove conclusively that nuclear DNA is the primary target of radiation. The need remains. Recently, space radiation protection and guidelines noted a need for more data collection on subcellular target radiation effects, and for the validation of computational transport models [7,32,36].

### 2.4. DNA Repair Mechanisms

DNA comprises two strands of a sugar-phosphate backbone and is connected by four nitrogenous bases, or base pairs (bp) [2]. The order and pairings of the bases and these chains of molecules determine the task for each cell and the overall genetic blueprint of the organism [50]. AP, or apurinic and apyrimidinic, sites are where lesions are present and can hinder DNA replication and transcription processes [51,52]. Ionizing radiation interacting with an organism’s cellular structures commonly triggers a stress response in which the free radical production increases, altering the redox state, and/or cellular homeostasis [53]. The presence and elimination of free radicals are part of an organism’s normal biological function, even in the absence of irradiation. The chain reaction of free radical mechanisms converts nutrients into chemical energy. It maintains redox homeostasis, the cellular function of response and feedback, and is part of maintaining a physiologic steady state [53]. However, when radiation exposure triggers a stress response, tissue inflammation can occur to remove diseased and damaged cells and prompt tissue repair mechanisms. During prolonged, continuous exposure to ionizing radiation, the biochemical processes that maintain homeostasis can malfunction, depending on the dose rate. This altered cellular environment affects the subcellular response, leading to more than an acceptable amount of DNA misrepair, secondary oxidative stress responses, deficiencies in DNA repair enzymes, and mutations, and can ultimately result in cell death [53,54].

Changes to the cellular microenvironment from ionizing radiation interactions can damage subcellular structures like nuclear DNA by creating reactive oxygen species (ROS) or free radicals. ROS can be in the form of superoxide anion (O_2_^−^) and the hydroxyl radical (OH^−^), and subsequently form the hydrogen peroxide molecule (H_2_O_2_) [55]. ROS are involved in a cell’s normal function. Approximately 104 lesions per cell from endogenous ROS formed in normal cellular processes are expected to occur daily [56]. Ionizing radiation adds to the number of lesions present, or damage load, and at large doses may overwhelm the cell’s antioxidative defenses [57]. These free radicals can be created directly from ionizing radiation interactions and come from the cell’s response to repair the damage from those interactions. As the organism ages, the lesions present on the DNA strands may also accumulate, and if misrepaired, these damaged sites can lead to DNA mutations and dysregulated cellular function [58]. Cell survival experiments suggest that the cell’s repair mechanism’s effectiveness decreases with increasing LET [46]. At the same dose rates, high-LET particles cause more oxidative clustered DNA lesions than low-LET radiation sources, since there is a higher percentage of irreparable and more complex double-strand breaks present [59,60]. There are observed repair mechanisms supporting some of the postulated radiobiological models. Once a single-strand break (SSB) or double-strand break (DSB) has been created in the target, processes are simultaneously triggered to repair the breaks.

Between low- and high-LET damage to DNA, the resultant type of lesion will cause the cell to favor one repair pathway over the other [54]. Depending on the distance between lesions along the DNA strands, the resulting breaks are categorized as DSB or SSB. For example, the term “clustered DNA lesions” is ascribed to multiple damaged sites within 20 bp of each other; these can be produced via endogenous or exogenous sources, such as ionizing radiation, normal cell function, or chemical toxicity [58]. When such multiple damaged sites are bunched across a short length, they may be more difficult to fully and faithfully repair through homologous recombination repair (HRR) since more of the DNA sequence will likely be lost, making clustered DNA lesions the most lethal form of all DNA damage caused by ionizing radiation [61]. Nonhomologous end-joining (NHEJ) is suggested to be the primary repair mechanism for high-LET DNA damage [54]. DSBs induced by ionizing radiation have blunt double-strand ends or short single-strand ends, which can be repaired via NHEJ [62].

Compared to DSBs, SSBs with ample space between events have a higher chance of repair. The most common repair method for SSBs is base excision repair (BER), which is an epigenetic regulation of gene expression [63,64]. BER restores the complementary nature of the bases in opposite DNA strands and is the most versatile [51,52,61,65]. For a BER process to be successful, it needs to result in no significant change to the nuclear DNA strand’s radiosensitivity [56]. Low LET is more likely to cause such sparsely clustered lesions, and this type of damage can utilize either NHEJ or HRR, whereas high-LET interactions and damage tend to be more densely clustered and, therefore, more complex in their repair [54]. Notably, the impact of clustered damage and DSBs on the cell’s ability to proliferate depends on the efficacy of the cell’s repair mechanisms, the dose rate, and the type of radiation [46].

### 2.5. Mitochondrial DNA

Recent research suggests a link between mitochondrial DNA (mtDNA) and radiation effects, but the more commonly used radiobiological models like the LQ model do not take mtDNA into account [66]. Mitochondria play a significant role in cellular energy production and are involved in the cell’s metabolism and oxidative stress control, as well as cellular death [67]. The DNA within mitochondria, referred to as mitochondrial DNA, can affect the risk of certain cancers and neurological diseases, and negatively influence the aging process [68,69]. Its importance challenges the assumption that nuclear DNA is the only subcellular structure of Interest in radiobiological models [66]. The primary role of mtDNA is to prepare for oxidative phosphorylation, a more efficient metabolic state for generating cellular energy [69]. In cases of repair, the microenvironment of the mitochondrion differs from that of nuclear DNA [56,70]. It should also be noted that the metabolism of mitochondria has been implicated in bystander radiation effects, but more research is needed to confirm their direct link [66].

Different cell types can be more susceptible to oxidative damage. For example, mtDNA is more vulnerable to oxidative damage and will mutate at a greater rate than nuclear DNA when damaged. This is because their proximity to the electron transport chain increases the chance of accumulating toxic ROS [56]. When the cellular environment’s redox stasis is imbalanced with an increased ROS level, this can lead to mitochondrial dysfunction and trigger intra- and extracellular distress signaling [69]. Oxidative stress, cellular respiration levels, and the mitochondrion’s metabolism respond to the environment by undergoing a morphological change to regulate its repair. The mitochondrial double membrane can go through fission and fusion actions to restore its function. The fission process allows the isolation and separation of the damaged proteins within the organelle.

In contrast, the fusion process mixes partially and fully functional mitochondria to create more fully functional ones [69]. Because of the high consumption and production of oxygen species present compared to other cell types, neuronal and muscular cells are more susceptible to oxidative stress effects [57]. Tissues with a higher concentration of mtDNA are expected to have a higher sensitivity to oxidative damage. They are more likely to result in mutations and deletions involved in ATP production. Adenosine triphosphate, or ATP, is the molecule involved in cellular energy generation and in the production of RNA, or ribonucleic acid, which aids in carrying out instructions from nuclear DNA [57]. Accumulation of mtDNA mutations may be linked to neurodegenerative diseases, such as amyotrophic lateral sclerosis [57].

Since each cell has multiple copies of mtDNA, it was suggested that strand breaks might not affect overall mitochondrial function [71,72]. While not as thoroughly researched as nuclear DNA repair, mtDNA studies show similar repair mechanisms. The typical nuclear DNA DSB repair pathway NHEJ was undetectable in mammalian mitochondria, but microhomology-mediated end joining, or MMEJ, was active [73]. MMEJ is a DSB repair mechanism that employs microhomologous or similar short sequence base regions [74]. Ionizing radiation causes oxidative stress, leading to mtDNA mutations and deletions. It is suggested that the damaged molecules undergo degradation after a DSB in mtDNA. This will only happen if a small amount of mtDNA is damaged, because certain cell types can have up to thousands of mtDNA molecules and the loss of a few would not compromise function [75]. So, the outcome of compromised cellular operation due to mtDNA damage, as suggested, is unlikely but still possible.

The ISS is within the LEO and those aboard the station benefit from the added radiation protection that the Earth’s magnetosphere provides [76,77]. This study of the twins did not conclude that genes altered in-flight compared to pre-flight and post-flight samples, or had increased numbers of DNA damage response (DDR)—which describes the cell’s process to repair and replicate DNA and continue through cell-cycle checkpoints—pathways [77]. The study instead saw changes to mitochondria within the subjects. The levels of mtDNA present within the subject aboard the ISS were higher than the pre-flight and post-flight sampling [77]. There is a positive correlation between time spent on the ISS and the concentration of mtDNA in the subject’s blood sample. The presence of mtDNA within the blood is possibly linked to inflammation, a typical result of radiation exposure [78]. With a limited testing pool and bias toward Caucasian middle-aged men, the result of this 2019 study implies the effects of prolonged exposure to ionizing radiation. However, additional research is needed to conclude the causation of these physiological changes in post-flight samples.

As previously stated, there are differences in the microenvironments surrounding mtDNA and nuclear DNA, and in their composition and damage repair. Nuclear DNA is linked with histones and chromatin-associated proteins that are involved in scavenging free radicals [56,79]. Although mitochondrial repair proteins are imported from the nucleus, mtDNA strands lack these scavengers. Furthermore, mtDNA has a higher density of coding sequences related to ATP production, which, if altered, affects overall cellular function. The danger arises if a damaged genome results in impaired oxidative phosphorylation and defective ATP production [80]. With a decrease in ATP production comes an increase in ROS production, which can trigger and accelerate the progression of different mammalian diseases [81].

This damage to mitochondria and mtDNA can be more extensive than that seen in nuclear DNA [79]. Once oxidative stress damages mtDNA, it lingers much longer than nuclear DNA damage and is more destructive. As a result of these differences in the presence of oxidative stress response, BER is the primary repair process available for mtDNA [56]. Though this is a repair mechanism for SSBs, it can still remove and repair deaminated and oxidized DNA bases [82]. It excises smaller DNA lesions caused by stressors, but most lesions induced by ionizing radiation are larger double-strand lesions that are irreparable or very complicated to repair. Thus, the maintenance of mtDNA is vital because of the risks involved in untended mutations.

Furthermore, it has been shown that when the cytoplasm of a more complex mammalian cell is altered or damaged, it can cause changes in mitochondrial function [83]. The component primarily involved in the process of mitochondrial fission is dynamin-related protein 1 (DRP1). This protein activates the autophagy process of the cell, which is also oxyradical dependent [83]. This action, where the dysfunctional mitochondria are isolated and degraded by the autophagy process, is thought to protect surrounding structures from the subsequent effects of irradiated cytoplasm. Additional research is needed to confirm the roles each subcellular organelle and gene expression plays in radiation effects, since even cell cytoplasm alterations can damage the organism [83].

### 2.6. Epigenetics

Besides direct DNA damage, it is being increasingly recognized that radiation exposure can also affect DNA and histone modifications, i.e., methylation. These modifications, generally known as epigenetic, are the key regulators of the expression of genetic information. DNA methylation is the most studied epigenetic modification of DNA, where the methyl group is bonded to the fifth position of carbon in the process facilitated by the enzymes called DNA methyltransferases and methyl-binding proteins [84].

Evidence accumulated throughout the last few decades convincingly demonstrates the potential ionizing radiation has to affect DNA methylation patterns. In rodent models, whole-body exposure to either γ radiation or x-rays at doses of 1 Gy and above usually results in the loss of global DNA methylation in many organs and tissues within hours of irradiation [85,86,87]. This effect may persist, typically in target organs for radiation-induced carcinogenesis, i.e., in the hematopoietic system (hematopoietic stem and progenitor cells, thymus) and mammary gland [85,88]. Loss of global DNA methylation in other organs (i.e., muscle or lung) has been shown to have largely transitory effects [85,89].

It must be emphasized that the loss of global DNA methylation is generally accepted as a hallmark of cancer [90]. As persistent DNA hypomethylation after exposure to IR was observed mainly in target organs for radiation-induced carcinogenesis, this led to the hypothesis that IR, besides exerting its genotoxic potential, may also cause cancer via an epigenetic mode of action [85]. While this hypothesis has not been fully confirmed, several mechanisms closely associated with carcinogenesis provide strong support. For instance, it is generally accepted that loss of DNA methylation usually occurs from otherwise heavily methylated repetitive elements that cover up to two thirds of mammalian genomes [91]. DNA methylation serves as a key mechanism of transcriptional silencing for repetitive elements [92]. For instance, Long Interspersed Nucleotide Element 1 (LINE-1)—the most abundant repetitive element in mammalian genomes—is a retrotransposon whose 5′-UTR sequence is heavily methylated to prevent its aberrant transcriptional activity [93]. As it covers ~17% and 22% of human and mouse genomes, respectively, loss of methyl groups from its promoter can result in its aberrant expression and retrotransposon activity. The latter is exhibited as a random introduction of its copy elsewhere in the genome. Such aberrant LINE-1 activity can lead not only to genome amplification but also significantly increase probability of mutations, as LINE-1’s copy can be introduced within the open reading frame (ORF) of a gene, thus affecting its transcription [94,95].

Besides global DNA hypomethylation, gene-specific DNA hypermethylation can occur due to exposure to ionizing radiation. Such events, if located within the gene promoters, are usually associated with transcriptional silencing, as the acquisition of methyl groups within the transcription start sites precludes the binding of transcription factors in the initiation of transcription. Similar to global DNA hypomethylation, hypermethylation-induced silencing of tumor suppressor genes is frequently observed in many cancers, including lung cancer of workers occupationally exposed to radiation [96,97].

Interestingly, exposure to high-LET radiation often shows differential patterns of DNA methylation alterations. For instance, several studies demonstrated loss of global DNA methylation in cell culture after exposure to low mean absorbed doses of protons or 56Fe ions [98,99]. However, the results of the in vivo studies appear contradictory, as we and others observed global DNA hypermethylation that stemmed from both repetitive elements and genes [100,101,102].

Another interesting outcome of high-LET radiation exposure is persistent changes in DNA methylation observed in organs that are considered targets for radiation-induced degenerative disease rather than carcinogenesis. For instance, persistently (i.e., 3–9 months after irradiation) altered DNA methylation was reported in the lungs and hearts of experimental mice after exposure to low mean absorbed doses of protons or heavy ions. These results were observed in several independently conducted experiments utilizing different sources and doses/rates of high-LET radiation [100,102,103,104,105].

Contrary to expectations, high-LET-induced DNA hypermethylation of repetitive elements often resulted in paradoxical reactivation of LINE-1 elements [102,106]. It is plausible to hypothesize that the complex interplay between DNA methylation and histone modifications, where the latter may “overwrite” the silencing effects of the former, is responsible for this effect [107]. There is a shortage of knowledge regarding the effects that high-LET radiation exerts on histone modifications, and future research is warranted to explore this phenomenon.

The epigenetic effects of exposure to high-LET radiation are much more complex and less understood compared to the effects exerted by terrestrial ionizing radiation. Nevertheless, elucidating epigenetic reprogramming, its mechanisms, and its effects on gene expression offers multiple opportunities to better understand the long-term effects of such exposures. Another important implication of epigenetics in space biology is the potential to utilize the methylation status of selective LINE-1 elements as biomarkers for previous exposures. As evident from the discussion above, exposure to ionizing radiation (including high-LET radiation) leaves scars, not only as mutations and irreparable damage to DNA itself, but also as permanently present alterations of DNA methylation within repetitive sequences (i.e., within the promoter regions of LINE-1 elements). Importantly, these altered patterns of DNA methylation can be detected not only in experimental systems, but also in humans previously exposed to ionizing radiation [100,102,108].

Histone modifications are another epigenetic mechanism of control over the expression of genetic information. Histone proteins can form the structural unit of a nucleosome that may undergo more than ten modifications. Perhaps the most pertinent to radiation research is the rapid phosphorylation of Ser 139 on histone H_2_AX—an initial step in recognizing radiation-induced damage [109]. The methylation of lysine 9 on histone H3 (H3K9) and lysine 20 on histone H4 (H4K20) are well-known histone marks that are not only associated with transcriptional silencing. Nevertheless, they are also suppressed after exposure to terrestrial ionizing radiation, thus potentially allowing for easier access of repair complexes to the sites of DNA damage [110]. Unfortunately, there is a dearth of knowledge regarding the effects of space radiation on histone modifications.

## 3. Discussion

Radiosensitivity depends on cell type, genetic composition, the microenvironment of the cell, and the radiation type and timing [36]. Radiation interacting with subcellular structures has the potential to alter the stress response and radiosensitivity of the cell and tissue [111,112]. It should be further noted that the radiosensitivity of cells differs between the individual cell type and the tissue as a whole: generally, the tissue or organ has a lower radiosensitivity than the individual cell [36,53]. Modeling the results of radiation on individual cells produces results that are not representative of radiation effects on the whole-body scale. Therefore, the LQ model may be inadequate for describing the mechanistic properties of radiation-induced biological and biochemical effects on the tissue or organism level. Individual proliferating cells may follow the LQ model, but aggregate cell populations appear more radioresistant [36].

There are separate benefits to in vitro and in vivo studies. In vitro irradiations may elucidate the behavior of lesion formation along DNA strands, while in vivo or animal studies can better connect whole-organ effects with ionizing radiation exposure. A study published in 2007 following the occurrence of oxidative clustered DNA lesions and DSBs from Cesium-137 gamma rays and 56Fe (at approximately 1.046 GeV/nucleon) found that high-LET radiation was more likely to create DSBs than oxidative DNA lesions [59]. This study also concluded that low LET induced higher yields of DSBs and oxidative lesions than high-LET particles. The samples were placed in a solution to mimic the cell’s natural chemical microenvironment. Between the two radiation sources, 56Fe ions resulted in a longer delay in DSBs returning to background levels [59]. Oxidative clustered lesions in the DNA strands also had a longer repair delay than DSBs, averaging between four and five days. During the fourth and eighth days of the study post-irradiation, DSBs within the samples increased, which was potentially related to apoptotic DNA fragments from misrepairs or unsuccessful repairs [59]. Other studies conducted within the field of radiobiology also concluded similar findings that DNA clustered lesions from high-LET interactions may have a delay in their repair, a misrepair, or a less completed repair of their DSBs [54]. In one of these studies, the irradiated cells were human monocytes, similar to the simplistic cells previously mentioned: composed of cytoplasm, a nucleus with DNA, and lysosomes [59]. This specific study did not compare its results to the irradiation of a more complex mammalian cell and acknowledged that the presence of some of the much smaller or shorter DNA fragments may not be detectable.

The level of oxygenation within the cellular microenvironment affects the effectiveness of the bombarding radiation, a concept closely studied in the context of hypoxia and tumor radiation response [2]. A more oxygenated environment will produce more free radicals, which can cause damage and alterations to the structure, nuclear DNA, and function of the cell [112]. Oxygenations may not play the same role for normal tissue in the space radiation environment. Where a tumor may have a hypoxic center that becomes oxygenated through targeted radiotherapy treatments, astronauts receive whole-body, continuous doses from charged particles [9]. Additionally, cell populations have naturally differing oxygenation levels. For example, lung epithelium has a higher oxygen-rich microenvironment than cardiac myocytes [113,114]. Therefore, how these tissues react to ionizing radiation will also differ.

Animal models have been used alongside numerical and computational efforts to predominantly enhance the study of nuclear DNA repair mechanisms. To better understand how BER affects miscoding nuclear DNA repair, genetically modified β-null mouse cells are deficient at repairing methylation-induced DNA lesions and can be used to study the monofunctional alkylating agents responsible for transferring single alkyl groups and often result in DNA coding errors and strand breakage [51]. Methyl methane sulfonate (MMS) seen in these β-null cell types is a monofunctional alkylating agent, and the presence of MMS-induced damage, in partnership with defects in genetic BER process deficiencies, has been connected to disease phenotypes [51]. Furthermore, an additional study hypothesized that nuclear genetic protein mutations and a reduction in the BER enzymes present within the cell can cause a build-up of nuclear and mtDNA mutations and lead to neurodegeneration [57]. There have been additional studies investigating the hypothesized connection between BER misrepair and the occurrence of Alzheimer’s disease [115].

In 2016, an experiment compared the presence of cardiovascular disease (CVD) in in-flight and non-flight astronauts to that in C57BL/J6 mice irradiated with a simulated galactic cosmic ray spectrum [76]. It suggested a connection between radiation exposure beyond LEO and the development of CVD, such as occlusive arterial disease (e.g., myocardial infarction, stroke). A conclusive link could not be determined because of limitations within the study, including a small sample size, an unknown source responsible for the results, and the differences in dose rate between the irradiated mice and the nominal space radiation environment [76]. Few studies have been conducted following the development of nonmalignant disease occurrence using astronauts. In 2019, another study followed the physiological difference between two male monozygotic twins where one incurred a prolonged stay of 340 days aboard the ISS. The comparison between the two subjects suggested that longer-duration missions could result in changes to cardiovascular physiology and affect oxygen distribution within the body as a consequence, which may alter the resulting biological effects [77]. From the study’s DDR, it was assessed that chromosomal aberrations potentially pointed to telomere-related instability [54,77]. Telomeres are the subcellular structure responsible for maintaining genomic integrity and play a role in preventing DNA degradation and erroneous DDR [77].

Our understanding of how charged particles interact with cells has made significant advancements and has more recently been used in space exploration research [116]. Given the low dose, low dose rate, and complexity of the space environment, models with a strong biological mechanistic focus may be best suited for space radiation research, but the utilized models still center on nuclear DNA damage and repair, effects of misrepair aberrations, and cell death, which are all topics more suited for radiotherapy treatments. The simplicity of the LQ model makes its use attractively straightforward, and it implicitly takes into account biological and chemical mechanisms occurring during ionization radiation interactions. However, its simplicity limits its ability to explain or model the underlying mechanisms.

Looking to the more recently developed numerical models that aim to incorporate biochemical and biophysical aspects, there are still limitations with each of these emerging methods. The TLK model can better match experimental data, but its limitation lies in its focus on cell survival and nuclear DSBs. It is based on experimentally irradiated Chinese hamster ovarian (CHO) cell data. And while this model was in good agreement with single-dose-administered survival (as opposed to the continuous dose present in space) and DSB rejoining data for the CHO cells, there were inconsistencies in more variable dosages and radiation types when compared to other experimental data [42]. The MCDS model is able to take differing environments and radiation types into account, but the system is hypothesized to overestimate the number of DSBs and does not consider the bystander effect [45]. Even the LEM and MKM models that incorporate dosimetric concepts fall short compared to experimental results and do not work for all irradiated cell types [36]. Proponents of these early models stated that more survival curve analyses are necessary to prove that nuclear DNA is the primary target of radiation.

To have a better overlap between experimental results and model predictions, both need to explore the impact that other subcellular components such as mtDNA have on cellular function and viability. There is also a need to better understand how the differing repair mechanisms between the two types of DNA affect potential mutations in irradiated samples and individuals. The animal studies mentioned within this review looking into the efficacy of BER repair need additional exploration prior to inclusion in any clinical considerations involving DNA repair from spaceflight radiation exposures, and the direct role BER plays in disease prevention needs to be better defined [56]. Moreover, the LQ model and its evolutions, and much of the space radiation and radiotherapy foci, have been primarily developed to explain cell survival and circumvent modeling of nonmalignant disease outside of DNA strand breaks and misrepair. As a result, there is still uncertainty as to what role subcellular dysfunction plays in whole-body effects.

## 4. Conclusions

Understanding how cellular components are affected by changes to their microenvironment and their role in tissue and organ dysfunction following irradiation can advance state-of-the-art space radiation protection and heavy ion radiotherapy. Recent advances in computational physics and biological sciences have contributed to the collective effort to better understand irradiation effects on cells, but each numerical and computational model has limitations. Furthermore, they all focus on nuclear DNA damage and repair without much regard for other subcellular structures. Applying these models to other subcellular damage and effects has the potential to develop a predictive model for deterministic effects and subsequently accelerate and support heavy ion radiotherapy efforts.

Mechanistic mathematical modeling of radiation-induced nonmalignant diseases can help provide insight into interpreting relevant experimental results and possible quantitative predictions related to heavy ion treatment results. Current radiobiological models describing the irradiation of mammalian cells focus on cell survival, and few predictive models for radiation effects incorporate non-nuclear DNA damage and repair. These more advanced models, such as the MCDS and TLK models, can better explain stochastic effects (e.g., cancer occurrence) and omit supportive evidence for modeling deterministic diseases following ionizing radiation exposure. Radiobiological models, if actively used, are appropriate for the radiotherapy setting, where disease or tumor eradication is the focus, and there is less of a practice to use these models in predicting whole-body outcomes [32]. There is a lack of experimental data following prolonged whole-body radiation exposure or a proper model that can describe the probabilistic behavior of radiation effects. More computational research and experimental data would need to be procured to better compare the damage and repair associated with ionizing radiation in nuclear versus mtDNA.

Studies of radiotherapy patients, occupationally exposed individuals, and atomic bomb survivors have shown a trend between high doses of low-LET radiation and the occurrence of degenerative tissue effects [117]. These nonmalignant disease occurrences following HZE nuclei exposures, like those experienced in spaceflight, are less understood. This is partly because of the type of radiation found in space and the characteristics specific to the spaceflight environment (e.g., microgravity, artificial and confined environment, additional stressors, etc.) [117]. The prolonged high-LET radiation exposure that an astronaut may experience requires further study. Because of the long latency period of the effects, non-nuclear structures may play a more significant role in irradiation outcomes.

Future models should consider the occurrence of nonmalignant or noncancerous disease following prolonged exposure to the GCR spectrum. Most research following the conclusion that nuclear DNA is the primary target of ionizing radiation has overlooked the role of other damaged subcellular structures. Further investigation into radiation-induced damage and the response of cellular organelles other than nuclear DNA was conducted decades after the genesis of the LQ model. The study found that each organelle within its scope has shown sensitivity to radiation and has subsequent effects [112]. Therefore, it is unreasonable to omit the changes to their structures, intercellular spacing, and function from radiation-induced damage and only consider the nuclear DNA breaks and aberrations. The foundational numerical models are built on the hypotheses of Crowther and Lea, whose oversight in assessing the complexity of the mammalian cell should be re-evaluated. Nuclear DNA has been set as the primary target of interest, and there is a focus on how damage to this subcellular structure and its ability to repair affect cellular proliferation.

Furthermore, different aspects of the space environment, such as microgravity and spaceflight time, may affect the cell’s ability to repair damage and the severity of the damage, respectively [118]. Previously conducted research found that seven genes, likely related to neuronal and endocrine signaling, which affects longevity-regulating transcription factors and dietary-restriction signaling, were suppressed during spaceflight [83]. In vitro studies of cellular response to the space radiation environment found that the repair pathways of prokaryotic cells, like bacteria, and simplistic eukaryotic cells, like yeast, are not affected by microgravity. However, more complex eukaryotic cells like those studied from the Shenzhou-8 space expedition suggested an enhanced DDR under microgravity [15]. This study did not find a significant change between spaceflight duration and DDR. Each of these repair mechanisms contributes to the resulting cell survival curves seen in radiobiological models. More radiobiological data supported by animal testing and additional insight into the long-term effects of space radiation exposure could improve the current radiobiological models used within the clinic. This would be an improvement that would reflect the advancements made within the field and have cascading benefits to multiple disciplines concerned with radiation effects.

## Data Availability

No new data were created or analyzed in this study. Data sharing is not applicable to this article.
